# Social Return on Investment of *Coming to Our Senses*: A Mindfulness-Based Intervention for Improving Mental Health and Wellbeing of NHS Healthcare Workers in Wales

**DOI:** 10.3390/bs16020194

**Published:** 2026-01-29

**Authors:** Alexander T. Friend, Bethany Anthony, Rachel Granger, Iwan Brioc, Ned Hartfiel, Rhiannon Tudor Edwards

**Affiliations:** 1 Centre for Health Economics and Medicines Evaluation, College of Medicine and Health, Bangor University, Bangor LL57 2PZ, UK; alexander.friend@bangor.ac.uk (A.T.F.); rachel.granger@bangor.ac.uk (R.G.); ned.hartfiel@bangor.ac.uk (N.H.); r.t.edwards@bangor.ac.uk (R.T.E.); 2 Theatr Cynefin, Cardiff, CF14 0RU, UK; iwanbrioc@gmail.com

**Keywords:** economic evaluation, health economics, health promotion, mindfulness, mental health, social return on investment, NHS workers, wellbeing valuation

## Abstract

Tackling poor mental health and wellbeing among healthcare workers remains a high priority for the National Health Service (NHS). This study evaluated the social value of the *Coming to Our Senses* mindfulness-based programme, designed to support the mental health of workers in high-stress medical environments, for NHS healthcare workers in Wales. Respondents (N = 39) to an online survey were assessed for mental health social value at baseline and one-month follow-up using the Short Warwick–Edinburgh Mental Wellbeing Scale (SWEMWBS) and the Social Value Bank (SVB). Social return on investment (SROI) ratios were calculated by dividing the change in mental health social value, health resource service use, and productivity costs by the programme delivery costs. Social value generated per respondent was £1890.05 using SWEMSWBS and £5775.97 using SVB. Cost savings in health resource and productivity were £9.41 and £79.10 per respondent, respectively. The programme delivery cost was £463.63 per respondent. Overall, including sensitivity analysis, the programme yielded a positive SROI of £2.35–£4.27:£1 using SWEMWBS or £6.82–£12.65:£1 using SVB. These findings suggest that the *Coming to Our Senses* programme may be effective in generating positive social value by improving self-reported mental health and wellbeing among NHS healthcare workers in Wales.

## 1. Introduction

Global evidence reports that nearly half of healthcare workers experience anxiety or depression, burnout, and public hostility and that 70% feel their employer fails to provide adequate mental health support (Squires et al., 2025). In the UK, mental health and wellbeing among healthcare workers remain a high priority concern for the National Health Service (NHS) (B. Taylor et al., 2025). According to the NHS Staff Survey 2024 (N = 774,828) (NHS England, 2024), 42% of staff reported feeling unwell due to work-related stress, 34% found their work emotionally exhausting, and 30% experienced burnout. Additionally, over half (56%) of healthcare workers had gone into work in the past three months despite not feeling well enough to perform their duties (NHS England, 2024), indicating that healthcare workers who are experiencing poor mental health and wellbeing are likely to have decreased productivity, efficiency, and quality of care (Søvold et al., 2021). Poor mental health and wellbeing among healthcare workers is estimated to cost the NHS £12.1 billion annually through increased absenteeism, presenteeism, and the need for cover staff (Daniels et al., 2022). Prioritising healthcare worker mental health and wellbeing can therefore enhance employee satisfaction and support the delivery of high-quality patient care.

Mindfulness is the focused awareness of the present moment, involving attention to bodily sensations, emotions, thoughts, and the surrounding environment, approached with an equanimous attitude (Chems-Maarif et al., 2025). Mindfulness-based interventions are shown to improve mental health and wellbeing in healthcare workers, including reductions in stress, depression, and anxiety (Fatemi et al., 2024; Ghawadra et al., 2020; Klatt et al., 2022; Knudsen et al., 2023; Pérez et al., 2022; Sos & Melton, 2023; Strauss et al., 2021; H. Taylor et al., 2022; Yang et al., 2018). It is important that economic evaluations of mindfulness-based interventions capture the wider intangible societal benefits of improved mental health and wellbeing, beyond direct healthcare resource costs, using approaches such as social return on investment (SROI) cost–benefit analysis (Edwards et al., 2015). Currently, the only available literature investigating the economic evaluation of mindfulness interventions for healthcare workers has focused predominantly on financially accountable costs, such as fewer staff injuries requiring medical treatment and decreases in staff turnover (Singh et al., 2015, 2016a, 2016b, 2018). While financially accountable outcomes are important, these evaluations are limited in scope, as they do not incorporate the social value associated with improvements in mental health and wellbeing, including reduced stress, burnout, and compassion fatigue. Despite the growing evidence supporting mindfulness-based interventions in healthcare settings, no studies to date have conducted SROI analysis to capture the full economic and social value of improved mental health and wellbeing among healthcare workers.

The aim of the present study was to assess the economic costs and social value change of mental health and wellbeing using SROI analysis of a distinctive, multimodal mindfulness-based programme designed to improve mental health and wellbeing for NHS staff across Wales (Stevenson, 2023).

## 2. Materials and Methods

### 2.1. Ethical Approval

This study received ethics approval from the Bangor University Medical and Health Sciences Academic Ethics Committee (application reference: 0643, approval date: 20 February 2025) and was conducted according to the guidelines of the Declaration of Helsinki, except for registration in a database, with written informed consent obtained from all participants.

### 2.2. Study Design

This mixed-methods study employed a natural experiment design within a real-world setting, in which the study was not deliberately designed by the researchers, and no control group was included. Economic evaluations embedded within natural experiments are increasingly recognised as both necessary and methodologically appropriate for investigating the socioeconomic determinants of health (Deidda et al., 2019; Edwards & McIntosh, 2019). The *Coming to Our Senses* programme was delivered as an open-invitation course to NHS employees in Wales, and participants signed up voluntarily. Participants’ health service resource use, productivity costs, and mental health and wellbeing outcomes were collected using an online pre–post survey at baseline and one-month follow-up after programme completion. The online survey study was administered via email by the course facilitator and was conducted between October 2024 and July 2025.

### 2.3. Sample

A total of 84 participants attended the *Coming to Our Senses* programme. Participants were recruited by an open-access approach with no formal entry requirements or selection process. Participants accessed the course primarily via self-referral through various advertisements of the programme by the project team via flyers, a pop-up information stand, and an intranet article. Eligible participants were NHS staff, aged 18 or over, who had the capacity to provide informed consent and were able to comprehend English to understand the mindfulness intervention and survey questionnaires. No additional exclusion criteria were applied. Survey participation was voluntary, and all participants who attended the programme were invited to take part in the survey at the first visit.

### 2.4. Coming to Our Senses Programme

The *Coming to Our Senses* programme offers a distinctive, multimodal approach to mindfulness-based interventions by embedding experiential methods drawn from applied theatre and group process. Unlike conventional mindfulness courses like Mindfulness-Based Stress Reduction or Mindfulness-Based Cognitive Therapy, each session is bookended by meditation practices and engages participants in Creative Challenges (interactive tasks), Journaling for Flow (expressive writing), and both theoretical and practical components that directly address contemporary mental health challenges. The course places equal emphasis on individual awareness and group dynamics, both essential pathways to improved mental wellbeing. The course encouraged participants to track their moment-to-moment experiences of stress, distraction, and uncertainty over time and to foster group bonding, mutual support, and compassion in a safe space for participants to share and express their discomfort.

An initial pilot (N = 14) of the *Coming to Our Senses* programme in healthcare workers at a single institute in Wales, UK, reported that 79% of respondents reported improved wellbeing, 71% treated themselves kindlier, and 57% reported improvements in work–life balance (Stevenson, 2023). Evaluation of the programme indicated that an investment of £3000 generated approximately £30,000 in social value (Stevenson, 2023).

Due to timetabling restrictions, the *Coming to Our Senses* programme was delivered in three models: Model A—eight 90-min in-person sessions; Model B—four 210-min in-person sessions fortnightly and three 90-min online sessions; Model C—four 210-min in-person sessions weekly. The intervention was delivered to eight cohorts at six sites: health board 1 (cohort 1.1 and 1.2, both Model B), health board 2 (Model C), health board 3 (Model A), health board 4 (cohort 4.1, Model A; cohort 4.2, Model C), health board 5 (Model C), and health board 6 (Model B).

### 2.5. Social Return on Investment Analysis

The SROI was calculated using two standardised perspectives of the HACT Mental Health Social Value Calculator (Trotter & Rallings Adams, 2017) and the HACT Social Value Calculator (Trotter et al., 2014). Both mental health valuations used in this study are grounded in wellbeing valuation and are a consistent and robust method for social cost–benefit analysis recommended in HM Treasury’s Green Book (HM Treasury, 2022). Monetized values of delivery costs, health service resource use, productivity costs, and mental health and wellbeing outcomes were used to calculate the social value of *Coming to Our Senses* for NHS employees in Wales.

#### 2.5.1. Delivery Costs

The costs of the *Coming to Our Senses* programme were calculated using a bottom-up, micro-costing approach using cost diaries of the costs associated with intervention delivery. Intervention delivery costs included intervention deliverer time, material preparation and creation costs, and venue hire and refreshment costs. For all attending staff, staff time was costed through multiplication of hours of training session with unit costs per working hour accounting for differing staff roles. All costs are presented in the British Pounds Sterling (£), rounded to the nearest whole pound.

#### 2.5.2. Health Service Resource Use

Resource use data was collected using a brief service use questionnaire. Unit costs were costed in (£) and were obtained from the Personal Social Services Research Unit (PSSRU) (Jones et al., 2025). Total health service resource use cost was calculated by multiplying the unit cost by the reported number of times each resource was used.

#### 2.5.3. Productivity Costs

The direct and indirect costs associated with employee workplace absenteeism and presenteeism were measured using the Institute for Medical and Technology Assessment (iMTA) Productivity Cost Questionnaire (iMTA Productivity and Health Research Group, 2020). To estimate the cost of absenteeism, the number of working hours missed during the past 4 weeks was multiplied by the standard cost price of productivity per hour. To estimate the cost of workplace presenteeism, the number of days worked while impaired was multiplied by one minus the efficiency score divided by 10 and then multiplied by the standard cost price of productivity per hour. The standard cost price of productivity was estimated as the gross hourly rate based on the occupational role details provided by participants.

#### 2.5.4. Mental Health and Wellbeing Outcomes

*HACT Mental Health Social Value Calculator*

The key outcome measure identified for the *Coming to Our Senses* programme was improved mental health and wellbeing measured using the Short Warwick–Edinburgh Mental Wellbeing Scale (SWEMWBS). SWEMWBS is a validated measure that asks respondents to rate their agreements with seven statements about different aspects of their mental health on a 5-point Likert scale, such as feeling confident, having a sense of purpose, enjoying life, and feeling optimistic (Stewart-Brown et al., 2009). Response scores to the SWEMWBS were summed (ranging from 7 to 35), with a higher score reflecting greater mental health and wellbeing. Specifically, a SWEMWBS score of 17 or less refers to ‘probable depression’, 18–20 refers to ‘possible depression’, 21–27 refers to ‘average mental wellbeing’, and 28–35 indicates ‘high mental wellbeing’. Wellbeing scores were categorised and assigned financial proxies from the Mental Health Social Value Bank (Table 1) (Trotter & Rallings Adams, 2017). If an individual moves from one SWEMWBS category score before an intervention to another SWEMWBS category score after the intervention, then the difference in corresponding monetary values reflects the economic value of increased or decreased mental wellbeing. Baseline and follow-up responses were compared for each participant to determine the number of participants who improved, stayed the same, or worsened for each outcome. To avoid over-claiming when utilising each HACT Mental Health Social Value Calculator, a standard deadweight percentage was subtracted from the calculated social value for each participant, reflecting the likelihood that some of the outcomes would have occurred even without the intervention.

*HACT Social Value Bank*

Mental health wellbeing questions from the Social Value Bank (Trotter et al., 2014) were also used to measure mental health, confidence, and perception of control over life, including ‘Do you suffer from depression or anxiety?’, ‘Have you recently been losing confidence in yourself?’, and ‘I feel that what happens to me is out of my control’. Social Value Bank questions include ‘valuable’ responses. If an individual moves from a response that is not marked as ‘valuable’ before the intervention into a ‘valuable’ response following the intervention, then the corresponding monetary value can be applied as a reflection of increased or decreased social value. The monetized values from the Mental Health Social Value Bank included £36,766 for relief from depressions/anxiety (mental health), £13,080 for high confidence (confidence), and £12,470 for feeling in control of life (perception of control over life). Baseline and follow-up responses were compared for each participant to determine who improved, stayed the same, or worsened. In addition to a deadweight adjustment to avoid overclaiming, attribution and displacement were included in the HACT social value bank SROI calculation. Attribution acknowledges that some outcomes may result from influences beyond the intervention itself, while displacement examines whether participants had to forgo other potentially beneficial activities. Discounting was not applied, as the time horizon of the analysis was limited to less than 12 months, and no costs or outcomes occurred beyond this period.

#### 2.5.5. Calculating the SROI Ratios

SROI ratios were calculated by dividing the social value generated from relevant outcomes per respondent by the total costs per respondent, expressed as the social value created per £1 invested in the intervention, using the following equation: SROI ratio = (social value change reported mental health and wellbeing outcome−deductions, i.e., % deadweight, % attribution, or % displacement)/(delivery costs + health service resource use costs + productivity costs). No discounting was included, as the time horizon was less than 12 months.

### 2.6. Budget Impact Analysis

The study findings and previously published statistics (StatsWales, 2025) were used in a budget impact analysis to estimate the potential financial impact of implementing the *Coming to Our Senses* programme across all NHS healthcare workers in Wales.

### 2.7. Open-Ended Questions

The participants had the opportunity to give qualitative responses regarding their own work-related mental health and wellbeing issues as well as expectations and feedback of the *Coming to Our Senses* programme. Specifically, respondents were asked two open-ended questions before starting the mindfulness intervention, i.e., “Are there any issues with stress, burnout, or mental health in a work context you would like to highlight?” and “Can you give a brief overview of what you are hoping to get from the course?”, and two open-ended questions after completion of the course, i.e., “Please comment on how helpful you found the course” and “Can you please comment why you would/would not recommend the course”. To allow themes to emerge inductively from the data, the open-ended responses were analysed using the framework approach (Ritchie & Lewis, 2003).

## 3. Results

### 3.1. Participant Demographics

The *Coming to Our Senses* programme recruited 84 participants, of which there were 54 (67.5%) respondents to the baseline survey and 39 (46.9%) respondents who also completed the follow-up survey (Table 2). Non-completion of the survey was attributable to voluntary non-participation, with no evidence of barriers to survey completion. Of those who responded to the baseline survey, 42 were female (78%), 8 were male (15%), and 4 (7%) preferred not to identify their gender. The mean age of a respondent was 46.5 ± 11.1 years, and the highest proportion of respondents identified their ethnicity as white (85%). Forty-three participants worked more than 30 h per week (80%), with the most common occupations including psychological and mental health (30%) and administrative, support, and business (20%). The details of occupational roles are detailed in Supplementary Table S1. Of those who responded to the follow-up survey, 11 respondents were delivered the *Coming to Our Senses* programme by Model A (28%), 13 respondents by Model B (33%), and 15 respondents by Model C (38%). Thirteen respondents attended all the sessions (33%), and more than half of respondents attended three-quarters of the sessions or more (N = 22, 56%). Respondents were released by their line manager (N = 24, 62%), attended the course in their own time (N = 14, 36%), or were given time off (N = 1, 3%). Reasons respondents were unable to attend sessions include prior or work commitments (N = 3), childcare (N = 1), travel time (N = 1), and technology issues (N = 1). Ten participants (19%) had accessed mental health or occupational health services within the last six months. Only the respondents who completed both baseline and follow-up questionnaires (N = 39) were included in the delivery costs, health resource use, productivity costs, mental health and wellbeing outcomes, and SROI analysis.

### 3.2. Social Return on Investment Analysis

#### 3.2.1. Delivery Costs

The cost of the total chargeable hours for the course was £16,296, which includes the administration tasks related to the preparation of the course, such as preparing course material, planning and rehearsing sessions, and post-session catch-ups and debriefs with co-facilitators, in addition to the delivery of the courses across the various models (Table 3). Total travel for all the sessions was estimated at 3968 miles, costing £1785 (costed at £0.45 per mile). In sum, the overall costs incurred for delivering the *Coming to Our Senses* programme was £18,081.60, yielding a cost of £463.63 per respondent (N = 39).

#### 3.2.2. Health Service Resource Use

The health resource unit count and use price per visit are detailed in Supplementary Tables S2 and S3. At baseline, the number of health service resource visits was 40 (mean cost £51.85 per visit, range £30.00–£171.00; Table 4), totalling an overall health service resource use cost of £2074.00 and yielding a cost of £53.18 per respondent. At follow-up, the number of health service resource visits was 34 (mean cost £50.21 per visit, range £30.00–£121.00), totalling an overall health service resource use cost of £1707.00 and yielding a cost of £43.77 per respondent. The overall cost saving in health service resource use costs was £9.41 per respondent.

#### 3.2.3. Productivity Costs

The standard cost price of productivity based on the estimated gross hourly rate of the occupational role provided by participants are detailed in Supplementary Table S4. At baseline, respondents were absent for a total of 125.8 h in the four weeks prior to the intervention, resulting in an absenteeism cost of £2456.34 (Table 5). During the same period, 114 workdays were affected by reduced productivity, with an average efficiency score of 3.4 ± 1.2, described as between a ‘slight’ and ‘notable’ impact on work, and was associated with a presenteeism cost of £10,155.28. At follow-up, respondents were absent for a total of 119.8 h in the four weeks following the intervention, resulting in an absenteeism cost of £1733.27. During the same period, 80 workdays were affected by reduced productivity with an average efficiency score of 3.5 ± 1.0, described as between a ‘slight’ and ‘notable’ impact on work, and was associated with a presenteeism cost of £7793.42. The overall cost saving in productivity costs was £79.10 per respondent.

#### 3.2.4. Deadweight, Attribution, and Displacement

The standard deadweight percentage for health interventions is 27%, as recommended in the established methodology (Trotter et al., 2014; Trotter & Rallings Adams, 2017). An estimated attribution percentage of 25% was applied to consider attributable factors other than the programme that could have contributed to an increase in outcome at follow-up independent of the intervention. Displacement was estimated at 5% since participants freely chose to participate in the programme and were not required to forego other activities from which they might have benefited (Hartfiel et al., 2023).

#### 3.2.5. Mental Health and Wellbeing Outcomes

The mean baseline SWEMSWBS score for the 39 respondents was 22.9 ± 4.0 and increased to 26.3 ± 3.9 following the *Coming to Our Senses* programme (Table 6). Thirty-two (82%) respondents reported an improvement in SWEMSWBS score by at least 1 point, with twenty-four respondents (62%) reporting an improvement in score of 3 or more and six respondents reporting an improvement in score of 7 or more (15%). This improvement in SWEMSWBS score corresponded to a positive change in social value of £73,711.98, adjusted for deadweight (27%), amounting to £1890.05 per respondent.

For the Social Value Bank questions, there was a net gain of nine respondents (23%) who reported a relief from depressions or anxiety, a net gain of four (10%) respondents who experienced an improved self-confidence, and a net gain of four (10%) respondents who experienced an improved perception of control over life (Table 7). The total resulting social value generated from the Social Value Bank, adjusted for deadweight (27%), attribution (25%), and displacement (5%), was £225,263.02, corresponding to £5775.97 per respondent.

#### 3.2.6. Social Return on Investment Ratios

SROI ratios were calculated for the two independent outcomes of mental health and wellbeing (SWEMWBS and Social Value Bank) by dividing the average financial value change per respondent by the average cost of delivery per participant (Table 8). Calculation of social value using SWEMWBS yielded a positive SROI return of £4.27: £1 (i.e., £4.27 of social value was created for every £1 invested in the intervention). Calculation of social value using Social Value Bank yielded a positive SROI return of £12.65: £1.

#### 3.2.7. Sensitivity Analysis: Consideration of Overheads and Venue Hire

Initial estimations of delivery costs (Table 3) and productivity costs (Table 5) were calculated without the inclusion of overheads. A sensitivity analysis was conducted considering the inclusion of an NHS institutional overhead (25%) and salary on-costs (32%, estimated based on the average on-cost from the PSSRU (Jones et al., 2025, p. 64)) for facilitator and respondent hourly wage. Further, costs of venue hire and refreshments were considered ‘in kind’ in the present programme, so an estimated cost of each was added in this sensitivity analysis. For delivery costs, venue hire cost was estimated at £35 per hour plus 30% institutional overhead (£4725.00), and refreshment cost was estimated at £2.50 per person per visit (£490.00). With the inclusion of overheads and salary on-costs to facilitator hourly rate, in addition to the venue hire and refreshments costs, the total intervention cost increased to £33,889.00 and intervention cost per respondent to £868.95 (Supplementary Table S5).

For productivity costs, the inclusion of NHS institutional overhead (25%) and salary on-costs (32%) increased absenteeism and presenteeism cost at baseline (£4359.78 and £18,023.13, respectively) and follow-up (£3075.98 and £13,831.25), leading to a greater overall savings (£5475.68) and savings per respondent (£140.40) (Supplementary Table S4). A second more conservative sensitivity analysis was conducted to match gross salaries taken from other sources (i.e., glassdoor.co.uk) for overheads detailed in the PSSRU (Jones et al., 2025) (i.e., non-staff overheads; management, administration, and estates staff overheads; capital overheads; and salary on-costs; Supplementary Table S4), and this led to lead to a greater savings per respondent (£223.90).

Applying the adjusted delivery and productivity calculations resulted in a decreased SROI for both SWEMWBS (£2.35–£2.44: £1) and Social Value Bank (£6.82–£6.92: £1) calculations compared to the base case analysis.

### 3.3. Budget Impact Analysis

To deliver the *Coming to Our Senses* programme to the entire NHS healthcare workforce in Wales (N = 112,135) (StatsWales, 2025) would cost between £52.0 million and £97.4 million (base case and conservative case, respectively) and generate between £222.0 million and £229.0 million (SWEMWBS) or £657.7 million and £664.5 million (Social Value Bank) in social value.

Of those who responded to the baseline survey (N = 54), 13 respondents were categorised to have possible depression (SWEMSWBS score <= 20). Considering the 24% prevalence of depressive symptoms among NHS healthcare workers in our cohort, it is estimated that 27,024 healthcare workers may be experiencing depressive symptoms. Targeting delivery of the *Coming to Our Senses* programme to those who may be experiencing depressive symptoms would cost between £12.5 million and £23.4 million (base case and conservative case, respectively) and generate between £53.5 million and £55.1 million (SWEMWBS) or £158.5 million and £160.2 million (Social Value Bank) in social value.

### 3.4. Open-Ended Questions

#### 3.4.1. ‘Are There Any Issues with Stress, Burnout, or Mental Health in a Work Context You Would Like to Highlight?’

Five key themes were highlighted with the largest two themes related to their job demands and the working organisation, structure, and environment. The first most common theme was issues relating to job demands, which included high workload and self-pressure, emotional and psychological stress of situations, and a reduced motivation and disengagement to work. The second theme was as equally as common related to their working organisation, structure, and environment and highlighted issues such as a lack of control in the way they work, a changing work environment, a lack of support and resources, and poor relationships with colleagues. Other key themes that emerged included juggling work with personal factors including home life, previous employments, and other health related issues; a lack of coping strategies to manage or resolve the stress, burnout, or mental health; and impacts on general health and wellbeing such as poor sleep and feelings of fatigue or pain. Further responses details for all open-ended questions are available in Supplementary Table S6.

#### 3.4.2. ‘Can You Give a Brief Overview of What You Are Hoping to Get from the Course?’

The majority of respondents hoped that the mindfulness course would help them acquire coping strategies to aid manage their work- and personal-related stress. Many hoped that the course would help them improve their emotional regulation and wellbeing, including relaxation, calming, and improvements in sleep, confidence, and positivity. Many also hoped that the course would provide them with the skills to improve their self-care, self-prioritization, and self-awareness by focusing on oneself and one’s own needs, to reconnect with or reset oneself, be kinder to oneself, and to remove expectation and gain perspective. Other aspirations relating to the intervention that emerged included an opportunity for creative exploration for those who had not experienced mindfulness before or further development skills they learned from previous mindfulness strategies and also meeting other participants for social connection and/or learning mindfulness strategies to support friends and colleagues.

#### 3.4.3. ‘Please Comment on How Helpful You Found the Course’

Respondents found the course to be very helpful. Specifically, respondents mentioned a variety of benefits to their personal development and wellbeing, including learning valuable techniques and tools to control mental health in work and outside of work, with several respondents beginning to implement these tools in their day-to-day life. Respondents reported that the course was well structured, with a good balance of challenging and supportive sessions and clear instruction from excellent teachers who guided them through the course. Some respondents mentioned how refreshing and innovative the course was in comparison to other courses or self-help sources they had tried. Respondents also enjoyed the group-working aspects of the course and sharing experiences with each other. One respondent did mention that some activities were challenging and they were not prepared to uncover psychological or physical issues and did not feel there was an option not to take part. One respondent mentioned it was unfortunate that the person who developed the Flow parts did not attend until the final session and said that if it had been at the start of the course, it would have enhanced the journey for them.

#### 3.4.4. ‘If You Attended the Course, Would You Recommend This Course to Your Colleagues?’ and ‘Can You Please Comment Why You Would/Would Not Recommend the Course’

All but one of the respondents (38/39) answered that they would recommend the course to their colleagues. Respondents once again highlighted the personal benefits and wellbeing that they gained from the course and how well the course was structured, delivered, and facilitated by the organisers. Respondents found the course enjoyable, would repeat the course, and felt that anyone would benefit from taking part. Respondents described that the course was life-altering and revolutionary, that they had never done anything like it before, and will always remember it. One respondent was sceptical prior to undertaking the course but said that they came away with so much more. A few respondents mentioned how the course would help them in their healthcare profession and improve their relationships with colleagues. One respondent mentioned that it is ‘important to do a course to facilitate healthcare professionals and the work they do. For healthcare professionals to do their best, it is important to have the best health and wellbeing. The course provides tools to help move forward in personal and professional lives’. A couple of respondents explained some caution when attending the course, as it could lead to participants experiencing some emotional distress, especially if they have previous or underlying traumas, and for participants to be prepared to be challenged and comfortable in opening up.

## 4. Discussion

### 4.1. Overall Findings

This study found that the *Coming to Our Senses* programme for NHS Wales healthcare workers yielded positive SROIs between £2.35–£12.65: £1. Qualitative feedback of the *Coming to Our Senses* programme supported the positive SROI’s, with respondents reporting benefits to their professional and personal life.

Questionnaire retention rate was good, with 39/54 (72%) respondents completing the baseline and follow-up questionnaires. The Social Value Bank-based SROI of £6.82–£12.65: £1 is comparable to the return on investment observed in the pilot of the *Coming to Our Senses* programme delivered to a single cohort (~£10.00: £1) (Stevenson, 2023), and the SWEMSWBS-based SROI of £2.35–£4.27: £1 is in line with other mindfulness-based programmes using the same methodologies (Hartfiel et al., 2023; Makanjuola et al., 2022; Whiteley et al., 2025). The *Coming to Our Senses* programme demonstrated strong acceptability with respondents and employers. Nearly all respondents indicated they would recommend the course to colleagues, and most respondents were released by their line manager to attend the programme, indicating its value for both professional and personal development as a healthcare worker. Engagement in the *Coming to Our Senses* programme was high, with the majority attending at least three quarters of the sessions and a good respondent assessment retention rate between baseline and follow-up assessments. Thematic analysis of qualitative data further emphasised the *Coming to Our Senses* programme’s usefulness and benefit to personal wellbeing and development, with some respondents noting its potential to provide healthcare staff with tools to perform effectively, manage conflict, and foster more open and inclusive workplace environments.

### 4.2. Strengths of the Study and Weaknesses of the Study

To our knowledge, this is the first study to integrate the social value of self-reported changes in mental health and wellbeing into an economic evaluation of mindfulness for healthcare workers. Specifically, this evaluation adopted societal (mental health and productivity) and healthcare system (resource use) perspectives within a multi-evaluation approach, applying two recognised mental health valuation methods (SWEMWBS and the Social Value Bank), both grounded in wellbeing valuation and endorsed in HM Treasury’s Green Book as a robust approach to social cost–benefit analysis (HM Treasury, 2022). SROI evaluations have important limitations that must be acknowledged, as estimates are based on assumptions to assess attribution, deadweight, displacement, and financial proxies (Fujiwara, 2015; Maier et al., 2015); however, they remain useful for capturing how interventions generate societal benefits (i.e., improved healthcare worker wellbeing) that, in turn, could translate to tangible workplace cost-saving benefits (i.e., improving quality of care and patient satisfaction (Li et al., 2024)).

The *Coming to Our Senses* programme was delivered in three different models, ranging from weekly shorter sessions to intensive half-day formats and blended online and in-person options. Despite variations between delivery models in this study, all models produced positive mental health and wellbeing outcomes and favourable SROI ratios, supporting evidence that mindfulness-based interventions can be delivered and tailored to the practical constraints of different organisations without diminishing therapeutic effect (Ong et al., 2024; Sos & Melton, 2023; H. Taylor et al., 2022). This adaptability increases the feasibility of integration into workforce development programmes in healthcare and potentially other high-pressure professions.

The intervention was conducted without a control group, and the one-month follow-up period was too short to capture potential long-term effects of mindfulness on healthcare workers’ mental health and wellbeing. Future scale-up studies should adopt a randomised controlled trial design (e.g., with a wait-list control group) to establish causality and extend the duration of follow-up data collection and consider incorporating refresher or top-up sessions to examine the programme’s mid- to long-term effects on mental health and wellbeing. The sample size for this evaluation is small (N = 84 participants; N = 39 respondents) and consisted predominantly of middle-aged White females; however, the inclusion of a range of occupational roles and the demographic profile being broadly representative of NHS Wales staff (77% female; 80% White) (StatsWales, 2025) provides some reassurance regarding generalisability.

### 4.3. Comparison with Previous Literature and Implications for Future Research, Policy, and Practice

In contrast to the consistent positive SWEMWBS valuations across all cohorts, one cohort reported a negative social value when measured with the Social Value Bank, suggesting that the two approaches of social valuation may not reflect the same mental health outcomes. This finding supports the recommendation that these methods of social valuation should be applied and interpreted separately (Trotter & Rallings Adams, 2017). The SROI levels observed in this study (£2.35–£12.65: £1) align with previous economic evaluations of mindfulness interventions for people experiencing poor mental health, with reported returns ranging from £2.57–£4.67: £1 (Hartfiel et al., 2023) to £4.12–£7.08: £1 (Makanjuola et al., 2022) and £3.30–£4.70: £1 (Whiteley et al., 2025). Our findings also fall within a broader range identified in a recent scoping review of mental health-related interventions that reported SROI values between £0.79 and £28: £1 invested (Kadel et al., 2022). As delivery costs were calculated per respondent (N = 39) rather than per participant that undertook the programme (N = 84), the delivery cost per individual would effectively be halved (£215.26 vs. £463.63). Whilst determining whether the inclusion of the extra participants (N = 45) would sustain, increase, or decrease the generated social value is unknown, existing evidence on the positive effects of mindfulness-based interventions on mental health and wellbeing suggests that the SROI would likely increase. The demonstrated social return on investment of the *Coming to Our Senses* programme highlights its potential as a cost-effective way to prevent stress and burnout among healthcare workers, enhancing and complementing current occupational health and wellbeing provision.

This study makes a theoretical contribution to the economic literature by demonstrating the suitability of SROI for evaluating staff wellbeing interventions within the NHS. Unlike traditional economic evaluations that prioritise patient health outcomes, such as cost-effectiveness analysis, SROI captures non-market outcomes and enables the valuation of staff wellbeing. The findings provide novel evidence of the potential social value generated by investing in preventative mindfulness interventions for NHS staff. Against the backdrop of rising service demands, limited resources, and increasing workforce pressures, these findings are practically relevant for decision-makers seeking to justify investment in staff wellbeing interventions. From a workforce-driven NHS context, this SROI analysis provides theoretical implications of value creation that goes beyond patient care by considering outcomes central to staff wellbeing in a bid to reduce burnout and improve staff retention.

All eight cohorts demonstrated improvements in mental health and wellbeing, alongside beneficial qualitative outcomes, following participation in the *Coming to Our Senses* programme. The mental wellbeing gains observed in this study are consistent with previous findings showing that mindfulness-based interventions in healthcare settings can alleviate stress, anxiety, and burnout while fostering greater compassion towards oneself and others (Fatemi et al., 2024; Ghawadra et al., 2020; Klatt et al., 2022; Knudsen et al., 2023; Pérez et al., 2022; Sos & Melton, 2023; Strauss et al., 2021; H. Taylor et al., 2022; Yang et al., 2018). Notwithstanding this, this evaluation suggests that the *Coming to Our Senses* programme has applications that extend beyond the improvements in self-reported wellbeing. By embedding Creative Challenges, Journaling of Flow, and facilitated group process alongside formal meditation, the *Coming to Our Senses* programme illustrates how art, embodiment, and group dynamics can advance the field of mindfulness-based interventions and meet contemporary mental health needs. This pedagogical innovation may broaden the population that can benefit from mindfulness or provide an alternative programme to conventional mindfulness-based curricula to other organisations such as schools, community groups, and populations to whom conventional mindfulness may feel inaccessible.

## 5. Conclusions

These findings suggest that the *Coming to Our Senses* programme may be effective in generating positive social value by acutely improving self-reported mental health and wellbeing among NHS Wales healthcare workers. The study was limited by the small sample size, short follow-up period, and the lack of a control group. Consequently, the findings of this study should be interpreted cautiously and not generalised beyond the context of this study, and future research in this area may wish to adopt more robust study designs with larger sample sizes. Nevertheless, amid increasing pressures on NHS staff and rising levels of burnout, embedding mindfulness-based programmes within workforce wellbeing strategies could deliver long-term economic value and service sustainability.

## Figures and Tables

**Table 1:** SWEMWBS scores and corresponding monetary values.

SWEMWBS Score	Monetary Values
7–14	£0
15–16	£9639
17–18	£12,255
19–20	£17,561
21–22	£21,049
23–24	£22,944
25–26	£24,225
27–28	£24,877
29–30	£25,480
31–32	£25,856
33–34	£26,175
35	£26,793

**Table 2:** Participant demographics, occupation, and course attendance.

	Baseline Survey (N = 54)	N	%
Age (Mean (SD))		46.5 (11.1)	
Gender	Female	42	78%
	Male	8	15%
	Prefer not to say	4	7%
Ethnicity	White	46	85%
	Asian/British Asian	4	7%
	Mixed/multiple ethnic group	1	2%
	Prefer not to say	3	6%
Occupation	Psychological and mental health	16	30%
	Administrative, support, and business	11	20%
	Allied health professionals	8	15%
	Public health and population Health	7	13%
	Medical and clinical	7	13%
	Quality, safety, and service improvement	3	6%
	Education, training, and research	1	2%
	No response	1	2%
Employment	≥30 h per week	43	80%
	<30 h per week	11	20%
Health board (sign-up, N)	Health board 1.1 (15)	8	15%
	Health board 1.2 (7)	4	7%
	Health board 2 (15)	12	22%
	Health board 3 (11)	8	15%
	Health board 4.1 (10)	7	13%
	Health board 4.2 (2)	1	2%
	Health board 5 (12)	7	13%
	Health board 6 (12)	7	13%
Follow-up survey (N = 39)			
Course model attended	Model A (8 × 90-min in-person sessions)	10	26%
	Model B (4 × 210-min in-person and 3 × 90-min online sessions)	13	33%
	Model C (4 × 210-min in-person sessions)	16	41%
Attendance	I attended all the sessions	13	33%
	I attended three-quarters of the sessions or more	22	56%
	I attended around half the sessions	3	8%
	I attended a quarter of the sessions or less	1	3%
	I didn’t manage to attend any of the sessions	0	0%

**Table 3:** Intervention delivery costs.

Health Board	N	Model	Preparation (Hours)	Delivery (Hours)	Hours with Additional Facilitator	Total Chargeable Hours	Course Cost	Mileage Cost	Total Intervention Cost	Total Intervention Cost per Respondent
Overall	39		208	118	13.5	339.5	£16,296.00	£1785.60	£18,081.60	£463.63
Health board 1.1	4	Model B	26	18.5	3.5	48	£2304.00	£720.00	£3024.00	£756.00
Health board 1.2	4	Model B	26	15		41	£1968.00	£90.00	£2058.00	£514.50
Health board 2	9	Model C	26	14	3.5	43.5	£2088.00	£21.60	£2109.60	£234.40
Health board 3	4	Model A	26	12	1.5	39.5	£1896.00	£0.00	£1896.00	£474.00
Health board 4.1	6	Model A	26	12	1.5	39.5	£1896.00	£360.00	£2256.00	£376.00
Health board 4.2	1	Model C	26	14		40	£1920.00	£180.00	£2100.00	£2100.00
Health board 5	6	Model C	26	14	3.5	43.5	£2088.00	£54.00	£2142.00	£357.00
Health board 6	5	Model B	26	18.5		44.5	£2136.00	£360.00	£2496.00	£499.20

Model A, 8 × 90-min in-person sessions; Model B, 4 × 210-min in-person and 3 × 90-min online sessions; Model C, 4 × 210-min in-person sessions. Standard hourly rate was costed as £48 per hour. Mileage was costed at £0.45 per mile. No travel costs were associated with health board 3, as these sessions were conducted at same location as the sessions for health board 4.1 cohort. One 3.5 h session in health board 1.2 cohort was cancelled.

**Table 4:** Health resource use unit count by health board.

Health Board	N	Baseline		Follow-Up		Saving	Saving per Respondent
		Count	Total	Count	Total		
Overall	39	40	£2074.00	34	£1707.00	£367.00	£9.41
Health board 1.1	4	10	£599.00	5	£310.00	£289.00	£72.25
Health board 1.2	4	1	£45.00	3	£199.00	−£154.00	−£38.50
Health board 2	9	16	£846.00	12	£604.00	£242.00	£26.89
Health board 3	4	0	£0.00	2	£75.00	−£75.00	−£18.75
Health board 4.1	6	8	£354.00	6	£259.00	£95.00	£15.83
Health board 4.2	1	0	£0.00	0	£0.00	£0.00	£0.00
Health board 5	6	1	£53.00	2	£75.00	−£22.00	−£3.67
Health board 6	5	4	£177.00	4	£185.00	−£8.00	−£1.60

The health resource unit count and use price per visit are detailed in Supplementary Tables S3 and S4.

**Table 5:** Productivity costs.

Health Board	N	Baseline					Follow-Up					Savings	Savings per Respondent
		Hours Absent	Absenteeism	Days Impaired	Efficiency Score	Presenteeism	Hours Absent	Absenteeism	Days Impaired	Efficiency Score	Presenteeism		
Overall	39	125.8	£2456.34	114.0	3.4 ± 1.2	£10,155.28	119.8	£1733.27	80.0	3.5 ± 1.0	£7793.42	£3084.93	£79.10
Health board 1.1	4	0.0	£0.00	11.0	2.7 ± 0.6	£779.27	0.0	£0.00	0.0	- ± -	£0.00	£779.27	£194.82
Health board 1.2	4	0.0	£0.00	3.0	5.0 ± 1.4	£76.16	50.0	£641.03	0.0	- ± -	£0.00	−£564.87	−£141.22
Health board 2	9	0.0	£0.00	24.0	2.8 ± 0.8	£2160.51	24.0	£315.07	23.0	3.8 ± 1.3	£1623.20	£222.24	£24.69
Health board 3	4	0.0	£0.00	0.0	- ± -	£0.00	0.0	£0.00	0.0	- ± -	£0.00	£0.00	£0.00
Health board 4.1	6	111.0	£2227.57	20.0	4.3 ± 1.3	£2162.71	31.0	£479.18	25.0	3.0 ± 0.8	£3134.31	£776.79	£129.47
Health board 4.2	1	0.0	£0.00	5.0	3.0 ± -	£685.28	0.0	£0.00	12.0	4.0 ± -	£1413.43	−£728.15	-£728.15
Health board 5	6	0.0	£0.00	39.0	3.7 ± 1.2	£2985.54	14.8	£298.00	16.0	4.0 ± 1.0	£1205.29	£1482.25	£247.04
Health board 6	5	14.8	£228.77	12.0	2.7 ± 0.6	£1305.82	0.0	£0.00	4.0	3.0 ± -	£417.19	£1117.39	£223.48

The standard cost price of productivity based on the estimated gross hourly rate of the occupational role provided by participants is detailed in Supplementary Table S5. Efficiency scoring: 1, ‘I didn’t have any days where I suffered from physical or psychological problems’; 2, ‘I was able to do just as much as normal’; 3, ‘It had a slight impact on the amount of work’; 4, ‘It had a notable impact on the amount of work’; 5, ‘It had a significant impact on the amount of work’; 6, ‘I was not able to do much work’; 7, ‘I was not able to do any work’.

**Table 6:** Social value from SWEMWBS.

Health Board	N	Baseline SWEMWBS (Mean ± SD)	Baseline Social Value	Follow-Up SWEMWBS (Mean ± SD)	Score ≤−1	Score ≥1	Score ≥3	Score ≥7	Follow-Up Social Value	Social Value Change with Deductions	Social Value Change per Respondent
Overall	39	22.9 ± 4.0	£811,636.00	26.3 ± 3.9	6	32	25	6	£923,520.00	£73,711.98	£1890.05
Health board 1.1	4	22.8 ± 2.5	£85,779.00	26.3 ± 3.5	1	3	2	1	£95,680.00	£6523.03	£1630.76
Health board 1.2	4	25.5 ± 2.9	£95,642.00	29.0 ± 3.8	0	4	3	0	£99,533.00	£2563.49	£640.87
Health board 2	9	19.8 ± 4.2	£145,438.00	23.8 ± 4.1	2	7	5	3	£193,353.00	£31,567.60	£3507.51
Health board 3	4	26.5 ± 3.7	£97,250.00	27.5 ± 0.6	1	3	2	0	£99,508.00	£1487.63	£371.91
Health board 4.1	6	22.2 ± 2.2	£128,179.00	24.5 ± 3.4	1	5	2	0	£137,729.00	£6291.78	£1048.63
Health board 4.2	1	22.0 ± -	£21,049.00	25.0 ± -	0	1	1	0	£24,225.00	£2092.43	£2092.43
Health board 5	6	23.0 ± 4.3	£125,107.00	27.8 ± 4.2	0	5	5	2	£148,544.00	£15,440.88	£2573.48
Health board 6	5	24.4 ± 4.2	£113,192.00	28.4 ± 4.2	1	4	4	0	£124,948.00	£7745.15	£1549.03

Deductions: Deadweight = 27%. SD, standard deviation; SWEMWBS, Short Warwick–Edinburgh Mental Wellbeing Scale.

**Table 7:** Social value from Social Value Bank.

Health Board	N	*Net Change*: Improved Self-Confidence	*Net Change*: Improved Perception of Control over Life	*Net Change*: Relief from Depression or Anxiety	Social Value Change with Deductions	Social Value Change per Respondent
Overall	39	4	4	9	£225,263.02	£5775.97
Health board 1.1	4	1	3	2	£64,506.94	£16,126.74
Health board 1.2	4	1	3	1	£45,384.03	£11,346.01
Health board 2	9	0	−1	2	£31,759.87	£3528.87
Health board 3	4	0	−1	1	£12,636.96	£3159.24
Health board 4.1	6	2	0	1	£32,729.39	£5454.90
Health board 4.2	1	0	1	1	£25,608.87	£25,608.87
Health board 5	6	0	−3	0	−£19,457.88	−£3242.98
Health board 6	5	0	2	1	£32,094.83	£6418.97

Unit costs: improved self-confidence, £13,080; improved perception of control over life, £12,470; relief from depression or anxiety, £36,766. Deductions: deadweight = 27%; attribution = 25%; displacement = 5%.

**Table 8:** Social return on investment ratios.

Health Board	Total Cost of Delivery per Respondent	Health Resource Use Cost Saving per Respondent	Productivity Cost Saving per Respondent	SWEMWBS			Social Value Bank		
				SWEMWBS Social Value per Respondent	Total Financial Value	SROI Ratio (Financial/Delivery)	Social Value Bank Social Value per Respondent	Total Financial Value	SROI Ratio (Financial/Delivery)
Overall	£463.63	£9.41	£79.10	£1890.05	£1978.56	£4.27:£1	£5775.97	£5864.49	£12.65:£1
Health board 1.1	£756.00	£72.25	£194.82	£1630.76	£1897.82	£2.51:£1	£16,126.74	£16,393.80	£21.68:£1
Health board 1.2	£514.50	−£38.50	−£141.22	£640.87	£461.16	£0.90:£1	£11,346.01	£11,166.29	£21.70:£1
Health board 2	£234.40	£26.89	£24.69	£3507.51	£3559.09	£15.18:£1	£3528.87	£3580.46	£15.27:£1
Health board 3	£474.00	−£18.75	£0.00	£371.91	£353.16	£0.75:£1	£3159.24	£3140.49	£6.63:£1
Health board 4.1	£376.00	£15.83	£129.47	£1048.63	£1193.93	£3.18:£1	£5454.90	£5600.20	£14.89:£1
Health board 4.2	£2100.00	£0.00	−£728.15	£2092.43	£1364.28	£0.65:£1	£25,608.87	£24,880.73	£11.85:£1
Health board 5	£357.00	−£3.67	£247.04	£2573.48	£2816.86	£7.89:£1	−£3242.98	−£2999.60	−£8.40:£1
Health board 6	£499.20	−£1.60	£223.48	£1549.03	£1770.91	£3.55:£1	£6418.97	£6640.85	£13.30:£1

SROI, social return on investment; SWEMWBS, Short Warwick–Edinburgh Mental Wellbeing Scale.

## Data Availability

All data supporting the findings of this study are available within the article and its Supplementary Materials.

## Supplementary Materials

The following supporting information can be downloaded at https://www.mdpi.com/article/10.3390/bs16020194/s1: Table S1: Occupational roles; Table S2: Health resource use unit count by visit; Table S3: Health resource use evidence; Table S4: Estimated hourly rate of the occupational role; Table S5: Intervention delivery costs inclusive of venue hire, refreshment, and overheads costs; Table S6: Open-ended questions thematic analysis.

## Abbreviations

The following abbreviations are used in this manuscript:

iMTA	Institute for Medical and Technology Assessment
NHS	National Health Service
PSSRU	Personal Social Services Research Unit
SROI	Social return on investment
SVB	Social Value Bank
SWEMWBS	Short Warwick–Edinburgh Mental Wellbeing Scale

## References

[B1-behavsci-16-00194] Chems-Maarif R., Cavanagh K., Baer R., Gu J., Strauss C. (2025). Defining mindfulness: A review of existing definitions and suggested refinements. Mindfulness.

[B2-behavsci-16-00194] Daniels K., Connolly S., Woodard R., van Stolk C., Patey J., Fong K., France R., Vigurs C., Herd M. (2022). NHS staff wellbeing: Why investing in organisational and management practices makes business sense—A rapid evidence review and economic analysis.

[B3-behavsci-16-00194] Deidda M., Geue C., Kreif N., Dundas R., McIntosh E. (2019). A framework for conducting economic evaluations alongside natural experiments. Social Science and Medicine.

[B4-behavsci-16-00194] Edwards R. T., Bryning L., Crane R. (2015). Design of economic evaluations of mindfulness-based interventions: Ten methodological questions of which to be mindful. Mindfulness.

[B5-behavsci-16-00194] Edwards R. T., McIntosh E. (2019). Applied health economics for public health practice and research.

[B6-behavsci-16-00194] Fatemi J., Vagharseyyedin S. A., Askari-Noghani A. (2024). The impact of mindfulness-based stress reduction on workplace well-being and empathy levels among nurses working in psychiatric wards in Iran: A controlled trial. Issues in Mental Health Nursing.

[B7-behavsci-16-00194] Fujiwara D. (2015). The seven principle problems of SROI.

[B8-behavsci-16-00194] Ghawadra S. F., Lim Abdullah K., Choo W. Y., Danaee M., Phang C. K. (2020). The effect of mindfulness-based training on stress, anxiety, depression and job satisfaction among ward nurses: A randomized control trial. Journal of Nursing Management.

[B9-behavsci-16-00194] Hartfiel N., Gittins H., Morrison V., Wynne-Jones S., Dandy N., Edwards R. T. (2023). Social return on investment of nature-based activities for adults with mental wellbeing challenges. International Journal of Environmental Research and Public Health.

[B10-behavsci-16-00194] HM Treasury (2022). The green book: Central government guideline on appraisal and evaluation.

[B11-behavsci-16-00194] iMTA Productivity and Health Research Group (2020). Manual of the iMTA productivity cost questionnaire (iPCQ).

[B12-behavsci-16-00194] Jones K. C., Weatherly H., Birch S., Castelli A., Chalkley M., Dargan A., Findlay D., Gao M., Hinde S., Markham S., Smith D., Teo H. (2025). Unit costs of health and social care 2024 manual.

[B13-behavsci-16-00194] Kadel R., Stielke A., Ashton K., Masters R., Dyakova M. (2022). Social Return on Investment (SROI) of mental health related interventions—A scoping review. Frontiers in Public Health.

[B14-behavsci-16-00194] Klatt M., Westrick A., Bawa R., Gabram O., Blake A., Emerson B. (2022). Sustained resiliency building and burnout reduction for healthcare professionals via organizational sponsored mindfulness programming. EXPLORE.

[B15-behavsci-16-00194] Knudsen R. K., Ammentorp J., Storkholm M. H., Skovbjerg S., Tousig C. G., Timmermann C. (2023). The influence of mindfulness-based stress reduction on the work life of healthcare professionals—A qualitative study. Complementary Therapies in Clinical Practice.

[B16-behavsci-16-00194] Li L. Z., Yang P., Singer S. J., Pfeffer J., Mathur M. B., Shanafelt T. (2024). Nurse burnout and patient safety, satisfaction, and quality of care: A systematic review and meta-analysis. JAMA Network Open.

[B17-behavsci-16-00194] Maier F., Schober C., Simsa R., Millner R. (2015). SROI as a method for evaluation research: Understanding merits and limitations. VOLUNTAS: International Journal of Voluntary and Nonprofit Organizations.

[B18-behavsci-16-00194] Makanjuola A., Lynch M., Hartfiel N., Cuthbert A., Wheeler H. T., Edwards R. T. (2022). A social return on investment evaluation of the pilot social prescribing emotionmind dynamic coaching programme to improve mental wellbeing and self-confidence. International Journal of Environmental Research and Public Health.

[B19-behavsci-16-00194] NHS England (2024). NHS staff survey national results.

[B20-behavsci-16-00194] Ong N. Y., Teo F. J. J., Ee J. Z. Y., Yau C. E., Thumboo J., Tan H. K., Ng Q. X. (2024). Effectiveness of mindfulness-based interventions on the well-being of healthcare workers: A systematic review and meta-analysis. General Psychiatry.

[B21-behavsci-16-00194] Pérez V., Menéndez-Crispín E. J., Sarabia-Cobo C., de Lorena P., Fernández-Rodríguez A., González-Vaca J. (2022). Mindfulness-based intervention for the reduction of compassion fatigue and burnout in nurse caregivers of institutionalized older persons with dementia: A randomized controlled trial. International Journal of Environmental Research and Public Health.

[B22-behavsci-16-00194] Ritchie J., Lewis J. (2003). Qualitative research practice: A guide for social science students and researchers.

[B23-behavsci-16-00194] Singh N. N., Lancioni G. E., Karazsia B. T., Chan J., Winton A. S. W. (2016a). Effectiveness of caregiver training in Mindfulness-Based Positive Behavior Support (MBPBS) vs. Training-as-Usual (TAU): A randomized controlled trial. Frontiers in Psychology.

[B24-behavsci-16-00194] Singh N. N., Lancioni G. E., Karazsia B. T., Myers R. E. (2016b). Caregiver training in Mindfulness-Based Positive Behavior Supports (MBPBS): Effects on caregivers and adults with intellectual and developmental disabilities. Frontiers in Psychology.

[B25-behavsci-16-00194] Singh N. N., Lancioni G. E., Karazsia B. T., Myers R. E., Winton A. S. W., Latham L. L., Nugent K. (2015). Effects of training staff in MBPBS on the use of physical restraints, staff stress and turnover, staff and peer injuries, and cost effectiveness in developmental disabilities. Mindfulness.

[B26-behavsci-16-00194] Singh N. N., Lancioni G. E., Medvedev O. N., Myers R. E., Chan J., McPherson C. L., Jackman M. M., Kim E. (2018). Comparative effectiveness of caregiver training in Mindfulness-Based Positive Behavior Support (MBPBS) and Positive Behavior Support (PBS) in a randomized controlled trial. Mindfulness.

[B27-behavsci-16-00194] Sos T., Melton B. (2023). Comparison of mindfulness practices for effectiveness of stress and burnout reduction in healthcare staff. Journal of Holistic Nursing.

[B28-behavsci-16-00194] Søvold L. E., Naslund J. A., Kousoulis A. A., Saxena S., Qoronfleh M. W., Grobler C., Münter L. (2021). Prioritizing the mental health and well-being of healthcare workers: An urgent global public health priority. Frontiers in Public Health.

[B29-behavsci-16-00194] Squires A., Dutton H. J., Casales-Hernandez M. G., Rodriguez López J. I., Jimenez-Sanchez J., Saldarriaga-Dixon P., Bernal Cespedes C., Flores Y., Arteaga Cordova M. I., Castillo G., Loza Sosa J. M., Garcia J., Ramirez T., González–Nahuelquin C., Amaya T., Guedes Dos Santos J. L., Muñoz Rojas D., Buitrago-Malaver L. A., Rojas-Pineda F. J., Jones S. (2025). A descriptive analysis of nurses’ self-reported mental health symptoms during the COVID-19 pandemic: An international study. International Nursing Review.

[B30-behavsci-16-00194] StatsWales W. G. (2025). NHS staff characteristics.

[B31-behavsci-16-00194] Stevenson R. (2023). Evaluation of coming to our senses.

[B32-behavsci-16-00194] Stewart-Brown S., Tennant A., Tennant R., Platt S., Parkinson J., Weich S. (2009). Internal construct validity of the Warwick-Edinburgh Mental Well-Being Scale (WEMWBS): A Rasch analysis using data from the Scottish Health Education Population Survey. Health and Quality of Life Outcomes.

[B33-behavsci-16-00194] Strauss C., Gu J., Montero-Marin J., Whittington A., Chapman C., Kuyken W. (2021). Reducing stress and promoting well-being in healthcare workers using mindfulness-based cognitive therapy for life. International Journal of Clinical and Health Psychology.

[B34-behavsci-16-00194] Taylor B., Scobie S., Palmer B. (2025). What does the NHS staff survey tell us about the changing behaviours and motivation of health care staff?.

[B35-behavsci-16-00194] Taylor H., Cavanagh K., Field A. P., Strauss C. (2022). Health care workers’ need for headspace: Findings from a multisite definitive randomized controlled trial of an Unguided digital mindfulness-based self-help app to reduce healthcare worker Stress. JMIR MHealth and UHealth.

[B36-behavsci-16-00194] Trotter L., Rallings Adams M.-K. (2017). Valuing improvements in mental health: Applying the wellbeing valuation method to WEMWBS.

[B37-behavsci-16-00194] Trotter L., Vine J., Leach M., Fujiwara D. (2014). Measuring the social impact of community investment: A guide to using the wellbeing valuation approach.

[B38-behavsci-16-00194] Whiteley H., Lynch M., Hartfiel N., Cuthbert A., Beharrell W., Edwards R. T. (2025). Health economics-informed Social Return on Investment (SROI) analysis of a nature-based social prescribing craft and horticulture programme for mental health and well-being. International Journal of Environmental Research and Public Health.

[B39-behavsci-16-00194] Yang J., Tang S., Zhou W. (2018). Effect of mindfulness-based stress reduction therapy on work stress and mental health of psychiatric nurses. Psychiatria Danubina.

