# Supplementary material for: Social Return on Investment of Coming to Our Senses: A Mindfulness-Based Intervention for Improving Mental Health and Wellbeing of NHS Healthcare Workers in Wales
Source: Behav Sci (Basel). 2026 Jan 29;16(2):194. doi: 10.3390/bs16020194 (PMC12937790; doi:10.3390/bs16020194)
Supplement: Supplementary file 1 [file behavsci-16-00194-s001.zip › behavsci-4014234-supplementary.pdf]

Supplementary Materials:

Social return on investment of *Coming to Our Senses*: A mindfulness-based intervention for improving mental health and wellbeing of NHS healthcare workers in Wales

Alexander Friend[1], Bethany Anthony[1], Rachel Granger[1], Iwan Brioc[2], Ned Hartfiel[1], Rhiannon Tudor Edwards[1]

[1] Centre for Health Economics and Medicines Evaluation, College of Medicine and Health, Bangor University, Bangor, United Kingdom

[2] Theatr Cynefin, Cardiff, UK

Correspondence: Bethany Anthony, Centre for Health Economics and Medicines Evaluation, Ardudwy, Bangor University, Normal Site, Bangor, Gwynedd LL57 2PZ, UK. Email: b.anthony@bangor.ac.uk

Supplementary Table S1. Occupational roles

Category	Occupations
Psychological & mental health (16)	Clinical Psychologist (3), Assistant Psychologist (3), Psychologist (2), Behaviour Practitioner, Wellbeing Practitioner, Bereavement Counsellor, Cognitive Behavioural Therapist, Psychotherapist, Emotional Wellbeing Assistant, Senior EIPPs Practitioner, Art Psychotherapist
Administrative, support & business (12)	Business Support (2), Benefit Support Worker, Welfare Rights Manager, Finance Director, Community Complex Conditions Patient Experience Officer, Personal Assistant, Record Management Officer, Project Support Officer, Senior Administrator, Senior Support Officer, Coordinator
Allied health professionals (8)	Assistant Practitioner, Dietitian, Occupational Therapist, Deputy Head of Physiotherapy, Complementary Therapist, Art Workshop Facilitator, Physiotherapy Technician, Rehabilitation Coach
Public health & population health (7)	Workplace Health Adviser, Associate Director of Population Health, Public Health Practitioner, Revalidation Manager, Senior Public Health Practitioner, Environment Public Health Scientist, Smoking Cessation Advisor
Medical & clinical (7)	Pharmacist, Epilepsy Specialist Nurse, Clinical Nurse Specialist, Registered Nurse Adult, Radiographer, Nurse Assessor, Microbiologist
Quality, safety & service improvement (3)	Programme Manager, Service Improvement Manager, Quality, Safety, and Improvement Team
Education, training & research (1)	PhD student and Research Assistant

Supplementary Table S2. Health resource use unit count by visit

Visit	Baseline			Follow-up		
	Unit count	Unit cost	Total	Unit count	Unit cost	Total
GP	10	£45.00	£450.00	10	£45.00	£450.00
Nurse	8	£53.00	£424.00	2	£53.00	£106.00
Healthcare assistant	7	£30.00	£210.00	6	£30.00	£180.00
Pharmacist	11	£57.00	£627.00	7	£57.00	£399.00
Physiotherapist	0	£44.00	£0.00	5	£44.00	£220.00
Occupational health	0	£54.00	£0.00	0	£54.00	£0.00
Physician assistant	0	£68.00	£0.00	1	£68.00	£68.00
Paramedic in clinic visit	0	£57.00	£0.00	0	£57.00	£0.00
Breast clinic consultant	2	£103.00	£206.00	0	£103.00	£0.00
Diabetes consultant	1	£103.00	£103.00	0	£103.00	£0.00
Occupational health	1	£54.00	£54.00	0	£54.00	£0.00
Midwife	0	£52.00	£0.00	1	£52.00	£52.00
Surgeon	0	£111.00	£0.00	1	£111.00	£111.00
Dentist	0	£121.00	£0.00	1	£121.00	£121.00
Subtotal	40		£2,074.00	34		£1,707.00
Cost per visit			£51.85			£50.21
Cost per participant (N = 39)			£53.18			£43.77

Supplementary Table S3. Health resource use evidence

Resource Use	Unit Cost	Unit	Source
**On Questionnaire, Responses**			
General Practitioner (GP) clinic visit	£45	per visit	Costed lasting on average 10 minutes. Includes qualifications and direct care staff (PSSRU, 2025, p.72).
Nurse clinic visit	£53	per hr	Costed as a Band 5. Includes qualifications. (PSSRU, 2025, p.70).
Healthcare assistant	£30	per hr	Costed as Support and Outreach Worker (PSSRU, 2025, p.89) which has a similar salary £22,651 (Healthcare Assistants and other support staff from table 12.2.1 (p.112) £22,984) but includes other costs.
Pharmacist	£57	per hr	Costed as a Band 6 Community-based Pharmacist (PSSRU, 2025, p.64).
Physician Assistant	£68	per hr	Cost as a Band 7 Community Health Professional (PSSRU, 2025, p.64).
Physiotherapist	£44	per hr	Cost as a Band 5 Community Health Professional (PSSRU, 2025, p.64).
**Other Free Text Responses**			
Breast Clinic Consultant	£103	per hr	Cost as a Consultant: Medical. Includes qualifications (PSSRU, 2025, p.106).
Diabetes Consultant	£103	per hr	Cost as a Consultant: Medical. Includes qualifications (PSSRU, 2025, p.106).
Midwife	£52	per hr	Costed as a Band 5. Includes qualifications. (PSSRU, 2025, p.101).
Surgeon	£111	per hr	Cost as a Consultant: Surgeon. Includes qualifications (PSSRU, 2025, p.106).
Dentist	£121	per hr of patient contact time	Cost as an NHS Dentist - Performer only (PSSRU, 2025, p.77).

Unit costs presented in the table reflect the resource use reported by survey respondents. PSSRU, Personal Social Services Research Unit (Jones et al., 2025).

Supplementary Table S4. Estimated hourly rate of the occupational role

Category	Occupation (N)	Base case		Conservative Case 1		Conservative Case 2		Source
		Salary	Per hour	Total Salary Costs	Per hour	Total Salary Costs	Per hour	
Psychological & Mental Health (16)	Clinical Psychologist (3)	£47,336.00	£24.27	£74,317.52	£43.08	£106,873.00	£68.00	Cost as a Band 7 Community Health Professional (PSSRU, 2024, p.64).
	Assistant Psychologist (3)	£30,142.00	£15.46	£47,322.94	£27.43	£69,860.00	£44.00	Cost as a Band 5 Community Health Professional (PSSRU, 2024, p.64).
	Psychologist (2)	£47,336.00	£24.27	£74,317.52	£43.08	£106,873.00	£68.00	Cost as a Band 7 Community Health Professional (PSSRU, 2024, p.64).
	Behaviour Practitioner	£30,142.00	£15.46	£47,322.94	£27.43	£69,860.00	£44.00	Cost as a Band 5 Community Health Professional (PSSRU, 2024, p.64).
	Wellbeing Practitioner	£30,142.00	£15.46	£47,322.94	£27.43	£69,860.00	£44.00	Cost as a Band 5 Community Health Professional (PSSRU, 2024, p.64).
	Bereavement Counsellor	£39,133.00	£20.07	£61,438.81	£35.62	£89,215.00	£57.00	Cost as a Band 6 Community Health Professional (PSSRU, 2024, p.64).
	Cognitive Behavioural Therapist	£39,133.00	£20.07	£61,438.81	£35.62	£89,215.00	£57.00	Cost as a Band 6 Community Health Professional (PSSRU, 2024, p.64).
	Psychotherapist	£39,133.00	£20.07	£61,438.81	£35.62	£89,215.00	£57.00	Cost as a Band 6 Community Health Professional (PSSRU, 2024, p.64).
	Emotional Wellbeing Assistant	£30,142.00	£15.46	£47,322.94	£27.43	£69,860.00	£44.00	Cost as a Band 5 Community Health Professional (PSSRU, 2024, p.64).
	Senior EIPPs Practitioner	£47,336.00	£24.27	£74,317.52	£43.08	£106,873.00	£68.00	Cost as a Band 7 Community Health Professional (PSSRU, 2024, p.64).
	Art Psychotherapist	£47,336.00	£24.27	£74,317.52	£43.08	£106,873.00	£68.00	Cost as a Band 7 Community Health Professional (PSSRU, 2024, p.64).

Administrative, Support & Business (11)	Business Support (2)	£25,000.00	£12.82	£39,250.00	£22.75	£57,500.00	£36.51	Costed as average salary "NHS Business Support Officer" from glassdoor.co.uk accessed 29.07.2025
	Benefit Support Worker	£22,000.00	£11.28	£34,540.00	£20.02	£50,600.00	£32.13	Costed as average salary "NHS Support Worker" from glassdoor.co.uk accessed 29.07.2025
	Welfare Rights Manager	£42,000.00	£21.54	£65,940.00	£38.23	£96,600.00	£61.33	Costed as average salary "NHS Manager" from glassdoor.co.uk accessed 29.07.2025
	Finance Director	£87,000.00	£44.62	£136,590.00	£79.18	£200,100.00	£127.05	Costed as average salary "NHS Director" from glassdoor.co.uk accessed 29.07.2025
	Community Complex Conditions Patient Experience Officer	£29,000.00	£14.87	£45,530.00	£26.39	£66,700.00	£42.35	Costed as average salary "NHS Patient Experience Officer" from glassdoor.co.uk accessed 29.07.2025
	Personal Assistant	£24,000.00	£12.31	£37,680.00	£21.84	£55,200.00	£35.05	Costed as average salary " NHS Personal Assistant" from glassdoor.co.uk accessed 29.07.2025
	Record Management Officer	£20,000.00	£10.26	£31,400.00	£18.20	£46,000.00	£29.21	Costed as average salary "NHS Medical Records Clerk" from glassdoor.co.uk accessed 29.07.2025
	Project Support Officer	£27,000.00	£13.85	£42,390.00	£24.57	£62,100.00	£39.43	Costed as average salary "NHS Project Support Officer" from glassdoor.co.uk accessed 29.07.2025
	Senior Administrator	£24,000.00	£12.31	£37,680.00	£21.84	£55,200.00	£35.05	Costed as average salary "NHS Senior Administrator" from glassdoor.co.uk accessed 29.07.2025
	Senior Support Officer	£29,000.00	£14.87	£45,530.00	£26.39	£66,700.00	£42.35	Costed as average salary "NHS Senior Support Officer" from glassdoor.co.uk accessed 29.07.2025

	Coordinator	£25,000.00	£12.82	£39,250.00	£22.75	£57,500.00	£36.51	Costed as average salary "NHS Coordinator" from glassdoor.co.uk accessed 29.07.2025
Allied Health Professionals (8)	Assistant Practitioner	£30,142.00	£15.46	£47,322.94	£27.43	£69,860.00	£44.00	Cost as a Band 5 Community Health Professional (PSSRU, 2024, p.64).
	Dietitian	£30,142.00	£15.46	£47,322.94	£27.43	£69,860.00	£44.00	Cost as a Band 5 Community Health Professional (PSSRU, 2024, p.64).
	Occupational Therapist	£30,142.00	£15.46	£47,322.94	£27.43	£69,860.00	£44.00	Cost as a Band 5 Community Health Professional (PSSRU, 2024, p.64).
	Deputy Head of Physiotherapy	£53,824.00	£27.60	£84,503.68	£48.99	£120,841.00	£77.00	Cost as a Band 8a Community Health Professional (PSSRU, 2024, p.64).
	Complementary Therapist	£39,133.00	£20.07	£61,438.81	£35.62	£89,215.00	£57.00	Cost as a Band 6 Community Health Professional (PSSRU, 2024, p.64).
	Art Workshop Facilitator	£26,399.00	£13.54	£41,446.43	£24.03	£61,803.00	£39.00	Cost as a Band 4 Community Health Professional (PSSRU, 2024, p.64).
	Physiotherapy Technician	£26,399.00	£13.54	£41,446.43	£24.03	£61,803.00	£39.00	Cost as a Band 4 Community Health Professional (PSSRU, 2024, p.64).
	Rehabilitation Coach	£26,399.00	£13.54	£41,446.43	£24.03	£61,803.00	£39.00	Cost as a Band 4 Community Health Professional (PSSRU, 2024, p.64).
Public Health & Population Health (7)	Workplace Health Adviser	£39,133.00	£20.07	£61,438.81	£31.51	£89,215.00	£57.00	Cost as a Band 6 Community Health Professional (PSSRU, 2024, p.64).
	Associate Director of Population Health	£89,458.00	£45.88	£140,449.06	£72.03	£197,550.00	£125.00	Cost as a Band 8d Community Health Professional (PSSRU, 2024, p.64).
	Public Health Practitioner	£30,142.00	£15.46	£47,322.94	£24.27	£69,860.00	£44.00	Cost as a Band 5 Community Health Professional (PSSRU, 2024, p.64).
	Revalidation Manager	£39,133.00	£20.07	£61,438.81	£31.51	£89,215.00	£57.00	Cost as a Band 6 Community Health Professional (PSSRU, 2024, p.64).

	Senior Public Health Practitioner	£30,142.00	£15.46	£47,322.94	£24.27	£69,860.00	£44.00	Cost as a Band 5 Community Health Professional (PSSRU, 2024, p.64).
	Environment Public Health Scientist	£47,336.00	£24.27	£74,317.52	£38.11	£106,873.00	£68.00	Cost as a Band 7 Community Health Professional (PSSRU, 2024, p.64).
	Smoking Cessation Advisor	£26,399.00	£13.54	£41,446.43	£21.25	£61,803.00	£39.00	Cost as a Band 4 Community Health Professional (PSSRU, 2024, p.64).
Medical & Clinical (6)	Pharmacist	£39,133.00	£20.07	£61,438.81	£35.62	£89,215.00	£57.00	Cost as a Band 6 Community Health Professional (PSSRU, 2024, p.64).
	Epilepsy Specialist Nurse	£39,263.00	£20.13	£61,642.91	£35.74	£96,492.00	£56.00	Cost as a Band 6 Hospital Based Nurse (PSSRU, 2024, p.101).
	Clinical Nurse Specialist	£39,263.00	£20.13	£61,642.91	£35.74	£96,492.00	£56.00	Cost as a Band 6 Hospital Based Nurse (PSSRU, 2024, p.101).
	Registered Nurse Adult	£30,142.00	£15.46	£47,322.94	£27.43	£69,860.00	£44.00	Cost as a Band 5 Community Health Professional (PSSRU, 2024, p.64).
	Radiographer	£30,142.00	£15.46	£47,322.94	£27.43	£69,860.00	£44.00	Cost as a Band 5 Community Health Professional (PSSRU, 2024, p.64).
	Nurse Assessor	£39,263.00	£20.13	£61,642.91	£35.74	£96,492.00	£56.00	Cost as a Band 6 Hospital Based Nurse (PSSRU, 2024, p.101).
	Microbiologist	£47,336.00	£24.27	£74,317.52	£43.08	£106,873.00	£68.00	Cost as a Band 7 Community Health Professional (PSSRU, 2024, p.64).
Quality, Safety & Service Improvement (3)	Programme Manager	£56,000.00	£28.72	£87,920.00	£50.97	£128,800.00	£81.78	Costed as average salary "NHS Programme Manager" from glassdoor.co.uk accessed 29.07.2025
	Service Improvement Manager	£47,000.00	£24.10	£73,790.00	£42.78	£108,100.00	£68.63	Costed as average salary "NHS Quality Improvement Manager" from glassdoor.co.uk accessed 29.07.2025

	Quality, Safety, and Improvement Team	£47,000.00	£24.10	£73,790.00	£42.78	£108,100.00	£68.63	Costed as average salary "NHS Quality Improvement Manager" from glassdoor.co.uk accessed 29.07.2025
Education, Training & Research (1)	PhD student and Research Assistant	£25,000.00	£12.82	£39,250.00	£22.75	£57,500.00	£36.51	Costed as average salary "NHS Research Assistant" from glassdoor.co.uk accessed 29.07.2025

Base Case: Gross salary: Hourly rate was calculated assuming 37.5 working hours per week, 52 weeks a year. Conservative Case 1: Costings for glassdoor.co.uk salaries were adjusted for a standard 25% overhead cost and salary on-costs matched to PSSRU estimate (32%, (Jones et al., 2025)). The hourly rate was calculated assuming 37.5 working hours per week, 46 weeks a year. Conservative Case 2: Costings for glassdoor.co.uk salaries are matched to ratio of Wages/Salary to Non-staff Overheads (45%, of Wages/Salary), Management, admin, and estates staff overheads (30%), Capital Overheads (23%), and Salary On-Costs (32%) detailed in the PSSRU (total 230%). The hourly rate was calculated assuming 37.5 working hours per week, 42 weeks a year as detailed in the PSSRU.

Supplementary Table S5. Intervention delivery costs inclusive of venue hire, refreshment, and overheads costs

Health board	N	Delivery model	Preparation (hours)	Delivery (hours)	Hours with additional facilitator	Total chargeable hours	Course cost (inc. overhead & salary on-cost)	Venue Hire (inc. overhead)	Refreshment cost	Mileage cost	Total intervention cost	Total intervention cost per respondent
Overall	39		208	118	13.5	339.5	£26,888.40	£4,725.00	£490.00	£1,785.60	£33,889.00	£868.95
Health board 1.1	4	4x 3.5 hr & 3x 1.5 hr online	26	18.5	3.5	48	£3,801.60	£612.50	£40.00	£720.00	£5,174.10	£1,293.53
Health board 1.2	4	4x 3.5 hr & 3x 1.5 hr online	26	15		41	£3,247.20	£612.50	£40.00	£90.00	£3,989.70	£997.43
Health board 2	9	4x 3.5 hr	26	14	3.5	43.5	£3,445.20	£612.50	£90.00	£21.60	£4,169.30	£463.26
Health board 3	4	8x 1.5 hr	26	12	1.5	39.5	£3,128.40	£525.00	£80.00	£0.00	£3,733.40	£933.35
Health board 4.1	6	8x 1.5 hr	26	12	1.5	39.5	£3,128.40	£525.00	£120.00	£360.00	£4,133.40	£688.90
Health board 4.2	1	4x 3.5 hr	26	14		40	£3,168.00	£612.50	£10.00	£180.00	£3,970.50	£3,970.50
Health board 5	6	4x 3.5 hr	26	14	3.5	43.5	£3,445.20	£612.50	£60.00	£54.00	£4,171.70	£695.28
Health board 6	5	4x 3.5 hr & 3x 1.5 hr online	26	18.5		44.5	£3,524.40	£612.50	£50.00	£360.00	£4,546.90	£909.38

No travel costs were associated with Health board 3 as these sessions were conducted same location as the sessions for Health board 4.1 cohort. 1x 3.5 hr session in Health board 1.2 cohort was cancelled. Standard hourly rate was costed as £48 per hour. Mileage was costed at £0.45 per mile. Venue hire costed estimated at £35 per hr. Refreshment cost estimated at £2.50 per person per visit. Overhead costs (25%) and salary on-costs (32%) were applied where detailed.

Supplementary Table S6. Open-ended questions thematic analysis

Question	Count	Theme	Examples
Are there any issues with stress, burnout, or mental health in a work context you would like to highlight?	13	Workload and Job Demands	High workload / cannot cope with demands of job<br>Self-pressure<br>Burnout<br>Emotional and psychological stress of situations<br>Vicarious trauma<br>Maintaining quality of the job under pressure<br>Reduced personal accomplishments<br>Disengaged from work
	12	Working organization, structure, and environment	Changing work environment (including possible job loss)<br>No control in the way they do work / not valued correctly<br>Lack of support or information<br>Lack of resources<br>Not enough information / support<br>Lack of relationships with colleagues / feelings of isolation<br>Poor relationship with leadership<br>Poor relationships with colleagues, bullying by colleagues
	8	Juggling work with personal factors	External stresses (home / family / health) impacts work performance<br>External factors cause the individual to be too busy<br>Personal life affecting ability to switch off or cope<br>Multiple demands / juggling<br>Delayed stress recovery / overhang following a stressful period
	7	Lack of coping strategies	Unable to manage or resolve stress / burnout / anxiety<br>Little time to decompress / recover<br>"Can't switch off"

	4	Impact on Health and Wellbeing	Fatigue, depletion, overwhelmed, feeling drained<br>Fatigue, pain, poor sleep<br>Impacted / poor sleep
Can you give a brief overview of what you are hoping to get from the course?	32	Acquire coping strategies for stress/anxiety from work and personal factors	Help to manage wellbeing and cope with ongoing stresses<br>Learn techniques and strategies for coping with stress / work and personal<br>Find strategies to cope with anxiety<br>Learn some new skills to help me manage my stress<br>To learn practices for myself<br>General building of mindful habits in my life<br>Learning to implement more mindfulness into everyday life<br>A structured opportunity to practice mindfulness<br>Dealing with anxiety / stress more effectively<br>Some support in how to manage current life<br>To learn to cope with anxiety in the workplace<br>To cope with daily stressors more easily<br>Acquire tools and knowledge that will help myself and others
	18	Emotional regulation and wellbeing	Relaxation<br>Learn relaxation<br>To switch off / feel more rested<br>To have a calmer and understanding way of thought<br>Calming techniques<br>Ability to be calmer, regain control of emotional reactions<br>Improve sleep<br>Improve own wellbeing<br>Gain confidence<br>To become more assertive<br>To feel positive<br>Learn about oneself<br>Understand oneself<br>Learn to be present

			Learn to adopt acceptance<br>Seek clarity
	17	Self-care, self-prioritization, and self-awareness	Focus on self needs / reconnect with self<br>Prioritise oneself, focus on own needs<br>Focus on self, peace and improve wellbeing<br>More focused on self, motivated importance to self<br>Be kind and remove expectation from oneself<br>Reset oneself<br>Put oneself first<br>Gain perspective to take time off when not well<br>Not be so devastated when work does not go well<br>Less need to be resilient<br>Focus on present and calm
	7	Supporting others and social connection	To help others<br>Connect to others<br>Meet others, group benefit
	5	Creative Exploration & Mindfulness Development	Trying something creative<br>Try a creative approach<br>Explore sources of stress<br>Build on previous mindfulness strategies<br>Explore application of mindfulness<br>Reflect on current skills, deepen practices<br>Make use of available courses that are usually expensive and hard to come by<br>Build on previous mindfulness strategies
Please comment on how helpful you found the course	23	Personal Development and Wellbeing	Help to cope with distressing time in work<br>Help to let go of things<br>Gained insight<br>Developed self-awareness, managing pressure and stresses<br>Learnt valuable tools, techniques - calming, self-help<br>Improved relationship with self and others / family

			Added positivity to day<br>Lifted mood and out of isolation<br>Better understanding of what can / cannot control<br>Making time for oneself, learnt acceptance<br>Relax, reconnect, calmer<br>Implementing skills into own life and helping others
	18	Helpfulness/Usefulness	Very helpful<br>Extremely helpful<br>Incredibly helpful
	10	Course Structure and Experience	Amazing / Incredible / Fantastic / Superb course<br>Good balance of challenging and support<br>Clear instruction<br>Interesting to get a different perspective compared to other courses<br>Course presented innovative and fresh, in the way self-help had not<br>Meditations very good, useful<br>Great to engage with others in the group
	4	Teachers	Excellent teacher<br>Great teacher<br>Amazing facilitator<br>Guided very well
	2	Critical Feedback	Unfortunate that person who developed Flow parts didn't attend until the end<br>Found some activities challenging, uncovered psychological and physical issues, not prepared. Did not feel that there was an option not to take part
If you attended the course would you recommend this course to your colleagues?<br><br>Why, why not?	38<br>1	Yes<br>No	
	16	Personal Benefits & Wellbeing	Very beneficial tips for relaxation and meditation<br>Promotes positive well-being<br>Feel more positive, valuable, resilient<br>Opportunity to take time for oneself, nurture oneself so can help others<br>Tools to manage stress, anxiety, depression

			Learnt alternative way of looking at problems<br>Help reflect on stress and anxieties, help to understand little control you have, focus on what is important and within control<br>Think differently about oneself, course gives you the tools to aid your day-to-day management<br>Teaches useful strategies for mindfulness and calmness, manage stress and anxiety<br>Encourages you to look inward and recentre oneself for own wellbeing<br>Course provides skills and practices that it okay to stop<br>Useful to those wanting to learn new skills, mindfulness skills to deal with stresses<br>Meditation helps you concentrate on breathing, to re-focus, destress, re-calculate, and provides a space to think<br>Helps improve self-awareness, proper care of oneself, personal improvement<br>Encouraged to go outside of comfort zone, embrace creativity, think outside box<br>Found useful to aid juggle life's other commitments
	11	Structure, delivery and facilitation	Well-run, well-paced.<br>Excellent guide<br>Great course content, delivered well<br>Group leaders were brilliant<br>Leader was calm and knowledgeable<br>Nice to meet range of people participating<br>Allocated time to think, prioritise self, and learn self-care tools<br>A safe space
	10	General Praise	Enjoyable and would benefit others<br>Enjoyed the course<br>Would recommend<br>Techniques are good, meditations are invaluable<br>Would repeat experience in heartbeat<br>Life altering, have a deeper relationship with colleagues<br>Never done anything like it before, always remember it<br>Revolutionary to me, came with a lot of emotion

			Anyone would benefit<br>Useful for those not experienced in mindfulness before<br>Very sceptical prior, but come away with so much more
	3	Professional & Healthcare Context	Important to do a course to facilitate healthcare professionals and the work they do. For healthcare professionals to do their best, it is important to have the best health and wellbeing. The course provides tools to help move forward in personal and professional lives.<br>Helps those working with others, leading others<br>Helps recognise humanity in others and help with conflict management<br>Creates an open and inclusive environment with colleagues
	2	Risks	Not that I would not recommend, would rather say maybe. It should be pointed out that taking part in these sessions could lead to participants experiencing some emotional distress, especially if have previous or underlying traumas.<br>With that then explaining that some techniques can then help with these heightened emotions, and signposting available to those who need further support. None of this was given.<br>Be prepared to be challenged and open up and share
